# Spindle Cell Hemangioma in the Mucosa of the Upper Lip: A Case Report and Review of the Literature

**DOI:** 10.1155/2018/1370701

**Published:** 2018-03-26

**Authors:** Kazuhiro Murakami, Kazuhiko Yamamoto, Tsutomu Sugiura, Tadaaki Kirita

**Affiliations:** Department of Oral and Maxillofacial Surgery, Nara Medical University, Kashihara, Nara, Japan

## Abstract

Spindle cell hemangioma (SCH) is a unique benign vascular lesion. We present a case of SCH in the upper lip of a 41-year-old woman. A submucosal nodular mass 30 × 20 mm in size was observed in the left upper lip. The mass developed 5 years earlier and enlarged after repeated ethanol injections. The mass was elastic firm, mobile, bluish in color, and well demarcated in magnetic resonance imaging. Under the clinical diagnosis of hemangioma, surgical excision was performed under local anesthesia. Microscopically, the lesion was composed of irregular cavernous spaces and multiple solid cellular areas. Cavernous spaces were filled with a mix of erythrocytes and organizing thrombi. The solid areas showed proliferation of spindle-shaped cells arranged haphazardly or in short interlacing fascicles. Immunohistochemically, most cells strongly reacted with vimentin. CD31, CD34, factor VIII, smooth muscle actin, and Wilms tumor-1 reacted with endothelial cells lining the cavernous spaces. The cells within solid areas consisted of mixed cell population with variable reaction for the markers except for factor VIII. From these findings, the diagnosis of SCH was made. Two years after surgery, no recurrence was noted. A review of SCH in the head and neck region is made.

## 1. Introduction

Spindle cell hemangioma (SCH) is a unique vascular lesion, which almost exclusively affects the dermis and subcutaneous tissues of the distal extremities. Perkins and Weiss named a solitary single tumor SCH and multifocal lesions crowded within the same region “spindle cell hemangiomatosis” [1]. More than 200 cases have been reported in the English literature until 2017; however, only 12 cases have been reported to have occurred in the soft tissues of the head and neck region in detail [1–12].

We present an additional case in the mucosa of the upper lip of a 47-year-old woman and review of the literature on SCH of the head and neck region.

## 2. Case Report

A 41-year-old woman presented with a mass on the left upper lip and difficulty in pronunciation. The mass developed after she bit the upper lip 5 years earlier. The volume of mass was not reduced; however, the patient complained of pain. One year after the development, she visited an otolaryngologist. The mass was diagnosed as mucocele and aspirated. However, only blood was aspirated from this lesion, and the lesion's size was not reduced. Two years after aspiration, the size of mass increased, and she visited a plastic surgeon. The lesion was diagnosed as hemangioma by magnetic resonance imaging (MRI). Ethanol was injected into the lesion twice. Although the lesion was slightly reduced at the first injection, it did not change at the second. She consulted a dental clinic two years after the second injection and then was referred to our department.

**Figure 1:**
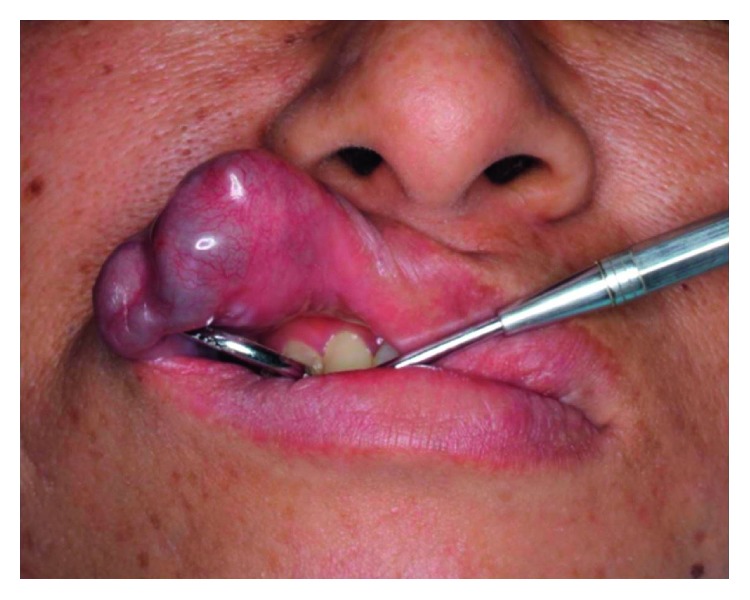
Clinical finding before surgery.

**Figure 2:**
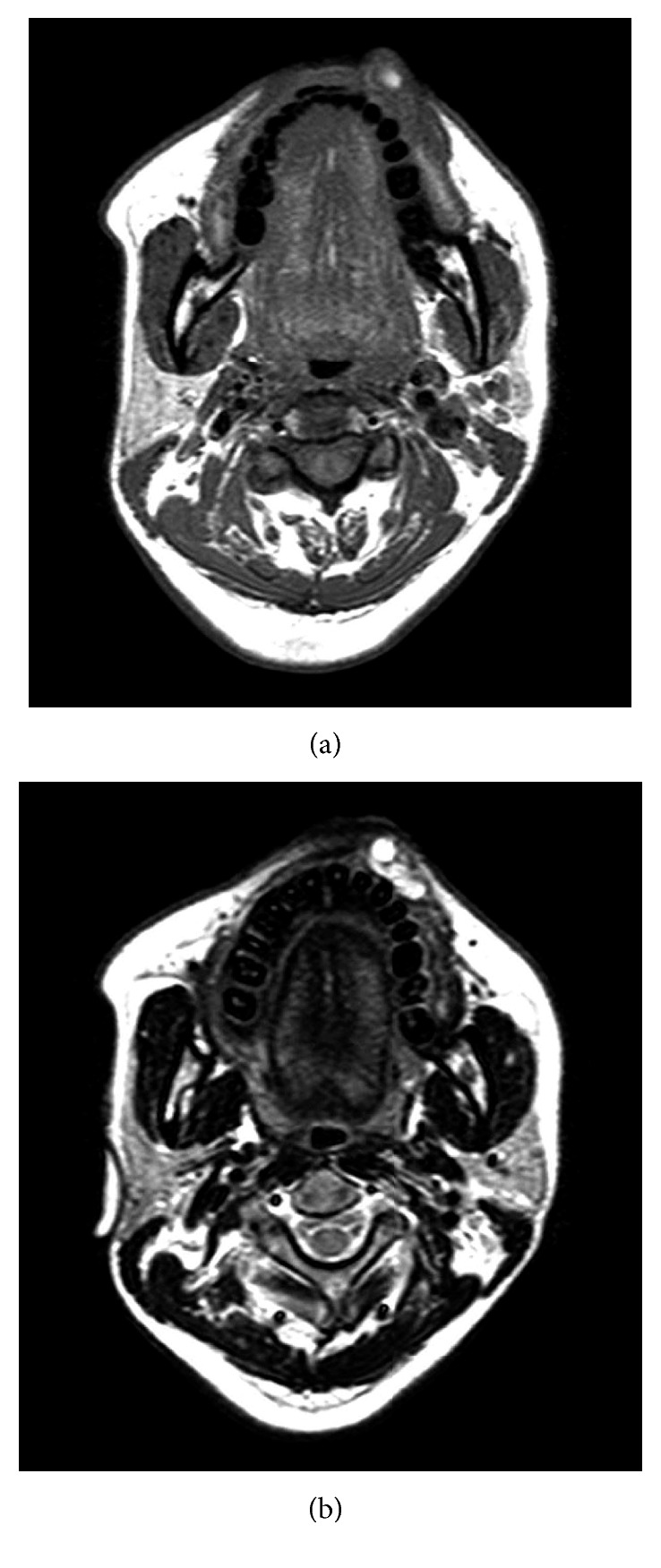
MRI findings. (a) T1-weighted image. (b) T2-weighted image.

**Figure 3:**
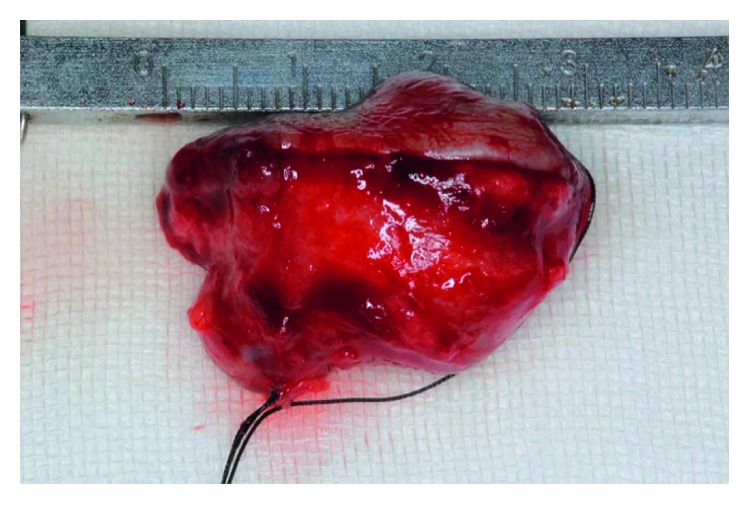
Excised tumor: lateral view.

**Figure 4:**
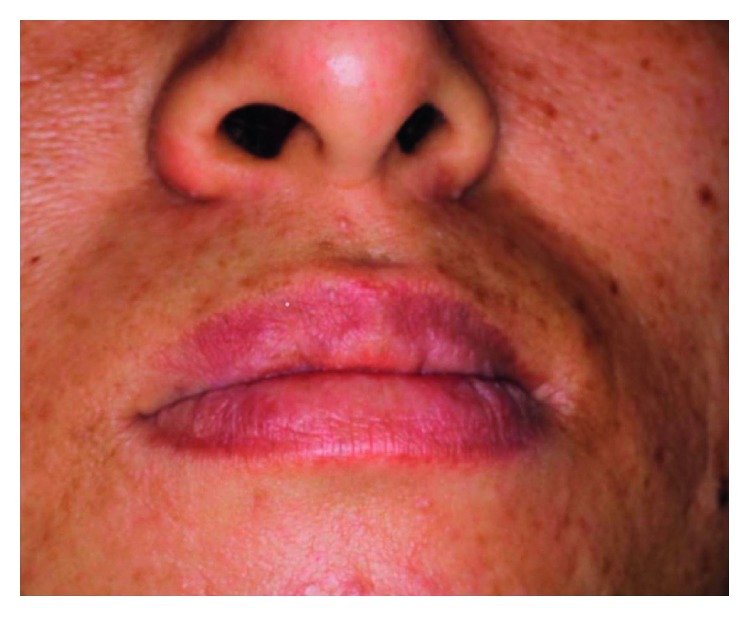
Clinical finding 6 months after surgery.

Oral examination revealed a circumscribed submucosal single nodule, approximately 30 × 20 mm in size in the left upper lip. The overlying mucosa was smooth, with bluish discoloration. On palpation, the nodule was elastic firm and mobile (Figure 1). Cervical lymph nodes were not palpable. MRI revealed a relatively well-demarcated lesion in the left upper lip. The lesion showed low signal intensity on T1-weighted images; however, it had a high signal area suspecting the subacute bleeding image in the centre of tumor. The lesion showed mostly high signal intensity on T2-weighted images (Figure 2). Under the clinical diagnosis of hemangioma, surgical enucleation was performed under local anesthesia. The tumor was removed with ligation and ablation of the inflow blood vessels. The overlying mucosa was partly removed, and the wound was closed by sutures (Figure 3). Postoperative course was uneventful. The patient was free of recurrence 2 years after surgery (Figure 4).

**Figure 5:**
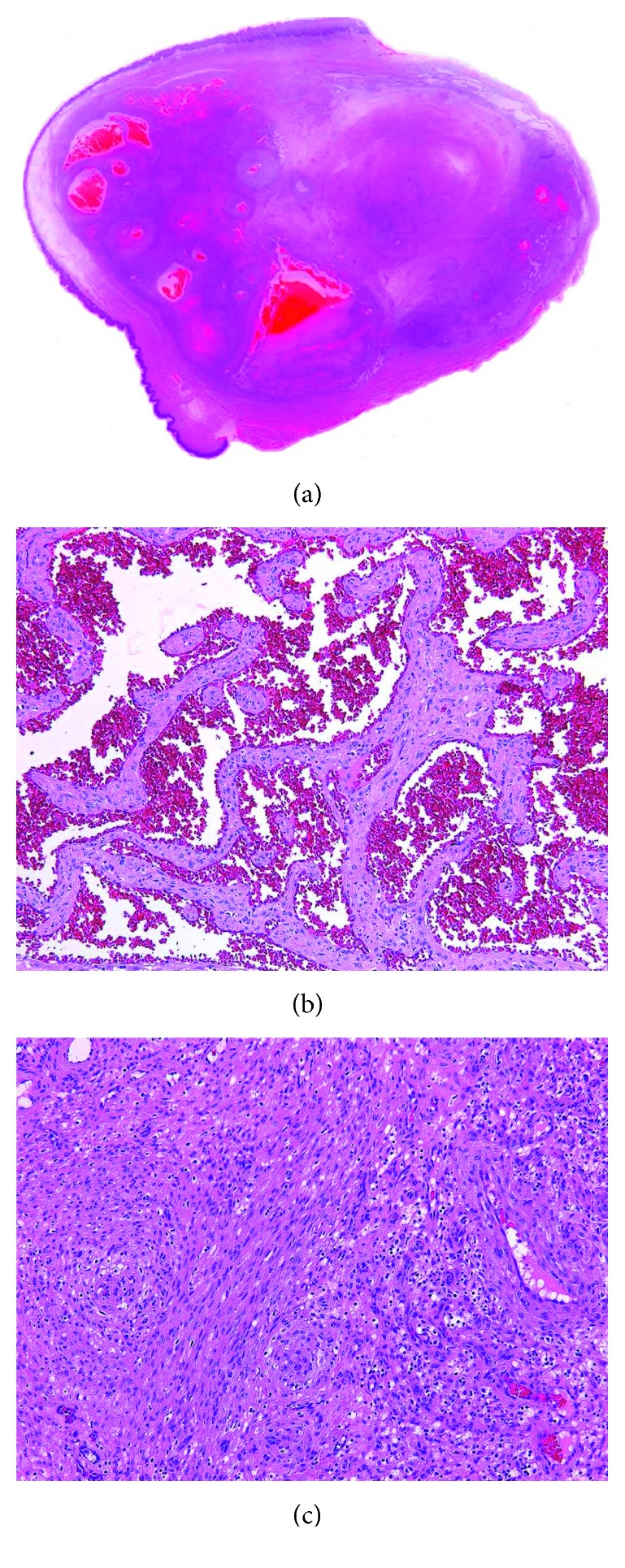
(a) Well-defined submucosal mass with cavernous spaces, solid areas, and number of thrombi. (hematoxylin and eosin stain, original magnification ×5). (b) Irregular cavernous spaces lined by flat endothelial cells. (hematoxylin and eosin stain, original magnification ×100). (c) The spindle-shaped cells in solid areas. (hematoxylin and eosin stain, original magnification ×100).

The size of excised the lesion was 30 × 20 mm. The specimen had reddish brown surfaces covered by thin-walled capsule including the mucosa of partial lower lip and inflow blood vessels which were well demarcated. Microscopically, the lesion was a well-circumscribed mass surrounded by fibrous connective tissue and showed a variety of cellularity imparting a lobular architecture in low power (Figures 5(a)–5(c)). The lesion was characterized by irregular cavernous spaces and solid cellular areas. The cavernous spaces contained erythrocytes and were lined by a single layer of flattened endothelial cells. Large cavernous spaces were filled with a mix of erythrocytes and organizing thrombi. The solid areas showed proliferation of spindle cells arranged haphazardly or in short interlacing fascicles. Epithelioid cells were also seen, some of which contained large cytoplasmic vacuole.

**Figure 6:**
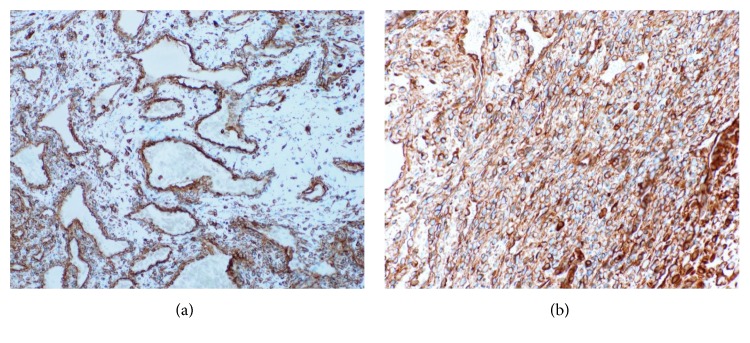
(a) Cavernous area (vimentin, original magnification ×100). Most endothelial cells around blood vessels in cavernous area strongly reacted. (b) Solid area (vimentin, original magnification ×200). Spindle cells and epithelioid in solid area are strongly positive.

**Figure 7:**
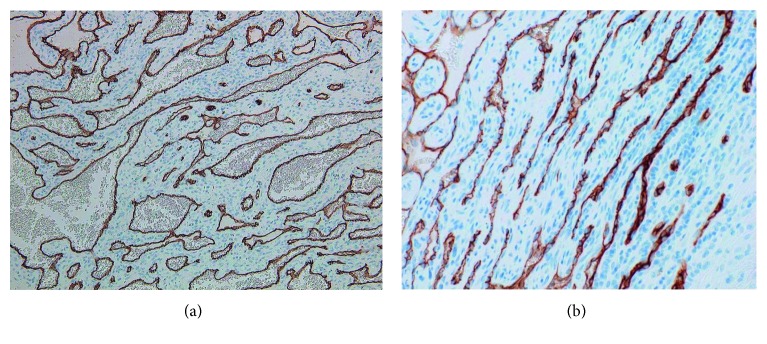
(a) Cavernous area (CD34, original magnification ×100). Most endothelial cells around vessels in cavernous space are positive. (b) Solid area (CD34, original magnification ×200). Spindle cells in solid area are focally positive.

**Figure 8:**
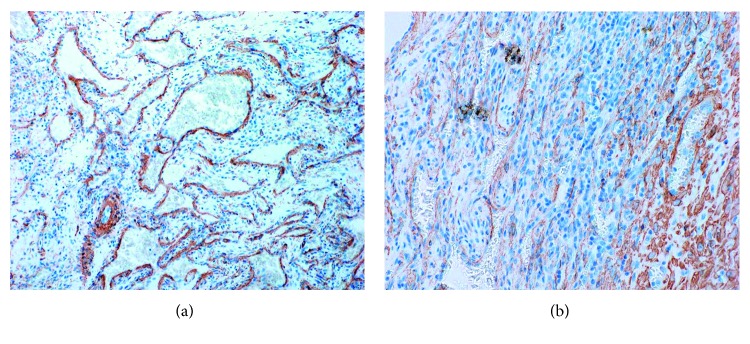
(a) Cavernous area (SMA, original magnification ×100). Most endothelial cells around vessels are positive. (b) Solid area (SMA, original magnification ×200). Spindle cells in solid area are focally positive.

**Figure 9:**
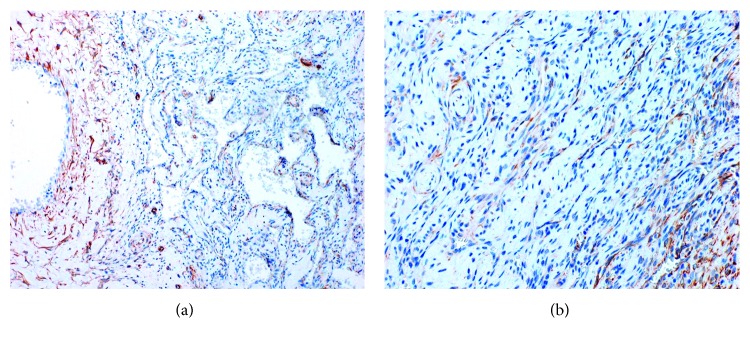
(a) Cavernous area (WT-1, original magnification ×100). Endothelial cells are strongly positive in cavernous area. (b) Solid area (WT-1, original magnification ×200). Spindle cells in solid area are focally positive.

Immunohistochemically, most endothelial cells lining the cavernous spaces, spindle cells within solid areas, and epithelioid cells within both areas strongly reacted with vimentin (Figures 6(a) and 6(b)). The endothelial cells lining the cavernous spaces reacted strongly with CD34 (Figure 7(a)), CD 31, factor VIII, smooth muscle actin (SMA) (Figure 8(a)), and Wilms tumor-1 (WT-1) (Figure 9(a)). The spindle cells within solid areas focally reacted with CD34 (Figure 7(b)), CD31, SMA (Figure 8(b)), and WT-1 (Figure 9(a)), whereas epithelioid cells were positive for SMA (Figure 8(b)), WT-1 (Figure 9(b)) and negative for CD34 (Figure 7(b)), CD31. S100 protein, AE1/AE3, D2-40, and EMA were negative in endothelial cells, epithelioid cells, and spindle cells. From these findings, the lesion was diagnosed as SCH.

## 3. Discussion

SCH has been diagnosed as various entities including mucocele, hemangioma, pyogenic granuloma, synovial sarcoma, and enchondroma (Table 1) [1–12]. In 1986, Weiss and Enzinger [13] described a unique vascular tumor as hemangioendothelioma with combined features of cavernous hemangioma and Kaposi's sarcoma. The tumor was considered to be an intermediate- or low-grade malignancy, with a biologic behavior between a hemangioma and an angiosarcoma. Fletcher et al. [14] proposed that hemangioendothelioma is caused by abnormalities of blood flow due to an arteriovenous shunt at the affected area, a view shared by some authors [15] and disputed by others [2]. Imayama et al. [16] suggested that hemangioendothelioma is a reactive process associated with vascular damage. They also hypothesized that the behavior of the endothelium may facilitate thrombosis and that a cyclical process of repeated thrombosis and thrombus organization with new vascular proliferation may explain the pathogenesis of the lesion. Later, Perkins and Weiss [17] proposed the terms SCH for solitary lesions and “spindle cell hemangiomatosis” for multifocal lesions. Since then, SCH has been used for solitary lesions.

Approximately 10% of cases are associated with other developmental anomalies or syndromes, including early-onset varicose veins, lymphedema, Klippel-Trenaunay-Weber syndrome, Maffucci syndrome, epithelioid hemangioendothelioma, and superficial cutaneous lymphatic malformations [8].

SCH is relatively uncommon. Only 12 cases have been reported in the soft tissues of the head and neck region (Table 1) [1–12]. Most cases were clinically diagnosed as intraoral vascular neoplasms, which are benign entities, including pyogenic granuloma, fibroma, peripheral giant cell granuloma, peripheral ossifying fibroma, inflammatory fibrous hyperplasia, and necrotizing ulcerative gingivitis [18]. In the majority of cases, the symptoms were not remarkable [18]. Most SCH lesions were fewer than 2 cm in size [3, 5, 7, 9, 11]. Only four cases in the head and neck region were more than 30 mm, including the present case, which was the only case of the oral lesion more than 30 mm. SCH may arise after trauma, such as repeated injections or surgical excision [17]. In the present case, the lesion developed by biting and enlarged by aspiration and repeated ethanol injection. MRI findings were consistent with those of any other hemangiomatous lesion. The lesion showed low signal intensity on T1-weighted images and high signal intensity in the majority of T2-weighted images. However, on T1-weighted image, high signal intensity was observed in the lesion's centre. The high signal intensity area is considered thrombi. The present case was diagnosed as hemangioma by clinical and MRI findings at first. The final diagnosis of SCH was made by histopathologic findings.

Histologically, SCH shows variable cellularity imparting a lobular architecture on low power. Large, ectatic, vascular spaces lined by flattened endothelial cells are frequently present. The cavernous spaces may contain calcified thrombi, referred to as phleboliths. Between the dilated vascular spaces, there is proliferation of spindle cells composed of endothelial cells, pericytes, and fibroblasts. The endothelial cells often have focal epithelioid features and show focal cytoplasmic vacuolization. Mitotic activity and atypia are low [11, 13].

SCHs resemble the features of Kaposi's sarcoma, such as male predominance and occasional multifocal growth. But Kaposi's sarcoma rarely contains cavernous vessels with thrombi and phleboliths and lacks epithelioid cells, and their spindle cells react for the endothelial marker CD34. On the other hand, SCH does not present the hyaline globules seen in Kaposi's sarcoma or express human herpes virus 8 latent nuclear antigen-1 [1, 17].

Immunohistochemically, the cells lining the cavernous space and vacuolated epithelioid cells are positive for vimentin, CD31, CD34, and factor-VIII-related antigen, supporting the endothelial nature [1–3, 5, 7–9, 11, 12, 15]. Weiss and Enzinger [13], Tosios et al. [1], Ide et al. [5], and Cai et al. [9] reported that the lining cells and vacuolated epithelioid cells are positive for vimentin, CD31, CD34, and factor VIII, and in contrast, spindle cells are negative for endothelial markers. However, Sheehan et al. [7] and Chavva et al. [8] reported that spindle cells are positive for these markers. In the present case, spindle cells are focally positive for these markers, (Table 2) suggesting that the tumor's solid area is composed of a mixed cell population with CD31-, CD34-, and factor-VIII-related antigen-negative cells as well as antigen-positive cells. SMA-positive pericytes reacted for both endothelial cells and spindle cells in all four reports including the present case which underwent this staining (Table 2). All cases which underwent staining with CD31, CD34, vimentin, and SMA (7, 8, 3, and 4 of 10 cases) were positive for endothelial cell, and all cases stained by vimentin and SMA were positive for both endothelial cells and spindle cells in (Table 2). Four of six cases stained by CD31 and five of seven cases stained by CD34 were positive for spindle cell. From these results, these markers were considered extremely sensitive markers in diagnosing SCH in the head and neck. S100, AE1/AE3, and EMA epithelial markers were negative in both cavernous and solid areas of two cases [9], including the present case; therefore, these makers may be not useful (Table 2). Tosios et al. [1] reported that CD 68, macrophage marker was positive; however, it was negative in the present case, and this marker's usefulness was not demonstrated (Table 2). Wang et al. [19] reported that SCH is a lymphatic malformation because D2-40 and Prox1 reacted in SCH. However, in the present case, D2-40 was negative, and Prox1 staining was impossible to conduct at our facility (Table 2). From this result, we could not determine if SCH is a lymphatic malformation. The proliferating epitheloid and round endothelial cells were negative for WT-1, a marker for differentiating vascular neoplasia from vascular malformation, whereas the spindle cells were focally positive in Wang's cases. However, in the present case, WT-1 was focally positive at both endothelial cells in cavernous areas and spindle cells in solid areas. Although Wang et al. suggested that SCH is primarily a lymphatic malformation, we consider that SCH is a vascular lesion arising from a vascular malformation, composed of mixed cells, such as vascular and lymphatic cells from the immunochemical result of all the markers performed in this case and other literatures [1, 5, 7, 9, 11, 15, 17, 19].

Surgical excision is the standard treatment for SCH, with excellent prognosis. Although more than 50% of patients may develop new lesions in the same anatomic region several years after initial excision [1], these are not considered a recurrence but new primaries or continuous multifocal intravascular growth. Postoperative radiotherapy and administration of low-dose interferon *α*-2b and recombinant interleukin-2 were reported to be successful in inaccessible lesions [1]. On the contrary, Chavva et al. [8] reported that radiation therapy is contraindicated due to the danger of malignant transformation. The literature contains no reports of patient mortality or metastasis from SCH. Spontaneous regression has been reported [14]. In the present case, the lesion was surgically removed, and no recurrence was observed 2 years after surgery.

## 4. Conclusion

SCH is considered an unusual vascular lesion in the oral cavity. Awareness of clinical, histologic, and histochemical features is important for differential diagnosis of the lesion from more aggressive vascular tumors to avoid unnecessary treatment. Although the common size of SCH is less than 20 mm, the size of it is guessed to be increased to more than 30 mm by frequent stimulation. In the diagnosis of SCH in head and neck, it is considered CD 31, CD 34, vimentin, and SMA are beneficial. We suppose the surgical removal of SCH is appropriate treatment with good clinical outcome.

## Figures and Tables

**Table 1:** Main clinical features of 13 cases of spindle cell hemangioma of the head and neck.

Author	Age	Gender	Duration (months)	Site	Maximum size (cm)	Clinical diagnosis	Follow-up (months)
Tosios et al. [1]	29	Female	12	Upper lip	1	Mucocele	36FOD
Scott and Rosai [2]	70	Male	RP	Ear	NA	Cavernous hemangioma	NA
Tosios et al. [3]	12	Female	NA	Mandibular vestibule	1	Hemangioma or pyogenic granuloma	LFU
Baron et al. [4]	1.5	Male	13	Lateral nasal side wall	1	Hemangioma	24 recurrence
Ide et al. [5]	55	Male	3	Palate	1.2	Pyogenic granuloma	12FOD
Lade et al. [6]	25	Male	6	Posterior pharyngeal wall	6	Synovial sarcoma	48FOD
Sheehan et al. [7]	44	Male	NA	Buccal mucosa	1	Vascular tumor	13FOD
Chavva et al. [8]	33	Male	8	Oral floor	1	Minor salivary gland tumor	6FOD
Cai et al. [9]	34	Female	24	Lower lip	2	Enchondromas (Maffucci syndrome)	NA
Minagawa et al. [10]	67	Female	4	Temporal muscle	4	Vascular tumor	24FOD
French et al. [11]	52	Female	6	Tongue	2	Polypoid haemangiomatous lesion	24FOD
Gbolahan et al. [12]	9	Female	12	Orbit	8	Tumor in the inferolateral orbit	21FOD
Present case	41	Female	60	Upper lip	3	Hemangioma	24FOD

RP: recent presentation; NA: not available; FOD: free of disease; LFU: lost to follow-up.

**Table 2:** Immunohistochemical analysis of the SCH cases in head and neck in the literature.

Author	CD34		CD31		VIMENTIN		SMA		Factor VIII		CD68		S100		EMA		AE1/AE3		D2-40		WT-1	
	C	S	C	S	C	S	C	S	C	S	C	S	C	S	C	S	C	S	C	S	C	S
Tosios et al. [1]	+	+					+	+	+	+	+	+										
Scott and Rosai [2]	+		+						+	+												
Tosios et al. [3]					+	+																
Ide et al. [5]	+	−	+	−			+	+	+	−												
Sheehan et al. [7]	+	+	+	+																		
Chavva et al. [8]	+	+	+	+																		
Cai et al. [9]	+	−	+	−	+	+	+	+					−	−			−	−	+	−		
French et al. [11]			+	+																		
Gbolahan et al. [12]	+	+																				
Present case	+	+	+	+	+	+	+	+	+	−	−	−	−	−	−	−	−	−	−	−	+	+

C: cavernous area; S: solid area; (+): immunopositivity; (−): immunonegativity.
